# Comparative developmental competence and gene expression in laparoscopically retrieved- and abattoir-derived goat oocytes

**DOI:** 10.3389/fvets.2026.1737059

**Published:** 2026-02-19

**Authors:** Abrar A. Malik, Riaz A. Shah, Syed Hilal Yaqoob, Mohammad Abrar Gayas, Nida Handoo, Suhail N. Magray, Mujeeb R. Fazili, Muneer A. Dar, Nafis I. Assad, Saba Nazir, Syed M. Shah, Nadeem Shabir, Syed M. Ahmad

**Affiliations:** 1 Embryo Biotech Laboratory, Division of Animal Biotechnology, Faculty of Veterinary Sciences, SKUAST-Kashmir, Srinagar, India; 2 Center of Excellence for Reproductive Biotechnology, Mountain Livestock Research Institute, SKUAST-Kashmir, Manasbal, India; 3 Department of Animal Husbandry, Govt of UT of Jammu and Kashmir, Srinagar, India; 4 Division of Teaching Veterinary Clinical Complex, Faculty of Veterinary Sciences, SKUAST-Kashmir, Srinagar, India; 5 Vaccine Biotechnology Laboratory, Division of Animal Biotechnology, Faculty of Veterinary Sciences, SKUAST-Kashmir, Srinagar, India; 6 Genomics Laboratory, Division of Animal Biotechnology, Faculty of Veterinary Sciences, SKUAST-Kashmir, Srinagar, India

**Keywords:** abattoir ovary, gene expression, goat, IVEP, LOPU, oocyte competence

## Abstract

**Introduction:**

Advancing *in-vitro* embryo production in goats requires identification of optimal oocyte sources and retrieval methods. While abattoir-derived ovaries provide abundant material for routine research, laparoscopic ovum pick-up from hormonally synchronized goats yields physiologically staged oocytes that may better support embryo development and advanced reproductive applications.

**Materials and methods:**

We compared the developmental competence and gene expression in bakerwal goat oocytes obtained via LOPU and from abattoir-sourced ovaries. A total of 528 cumulus oocyte complexes (COCs) were collected from 12 live donors across three LOPU sessions, while 1,517 COCs were retrieved from 338 abattoir ovaries. All oocytes underwent *in vitro* maturation, fertilization, and culture under similar conditions. Developmental competence was evaluated by cleavage, morula, blastocyst formation, and blastocyst morphology. Expression of competence-associated genes (*ZAR1, MFN2, BAX,* and *BCL2*) was quantified at immature oocyte, mature oocyte, and early blastocyst stages.

**Results:**

LOPU-derived oocytes demonstrated significantly higher developmental performance, including cleavage (54.9% vs. 45.2%), morula (45.9% vs. 34.9%), and blastocyst rates (29.5% vs. 19.0%; *p* < 0.05). Nuclear maturation did not differ significantly between groups (81.1% vs. 76.3%; *p* > 0.05). Gene expression analysis revealed enrichment of ZAR1 at GV and MII stages (up to 5.79-fold), progressive upregulation of MFN2 at the blastocyst stage (5.98-fold), downregulation of pro-apoptotic BAX (0.26–0.57 fold), and upregulation of pro-survival BCL2 (up to 1.85-fold) in LOPU-derived samples.

**Conclusion:**

In goats, oocytes retrieved via LOPU show superior developmental competence and more favorable molecular signatures than those obtained from abattoir-derived ovaries. LOPU-derived oocytes provide a more reliable option for achieving higher -quality embryos and may be more suitable for application in advanced reproductive biotechnologies.

## 1 Introduction

*In-vitro* embryo production (IVEP) provides a critical pathway for genetic improvement and conservation in domestic animals (1, 2), but its efficiency in goats remains low compared to other domestic animals, primarily due to inconsistent oocyte developmental competence (3). The developmental competence of an oocyte is governed by cytoplasmic processes, including maternal mRNA storage, mitochondrial redistribution, and apoptosis regulation (4, 5). Mammalian oocytes accumulate maternal mRNAs in a mitochondria-associated ribonucleoprotein domain. This domain stores translationally repressed mRNAs in a membraneless compartment around the mitochondria (6). Zygote arrest 1 (*ZAR1*), drives the assembly of this compartment apart from being a maternal-effect gene essential for oocyte maturation and embryonic genome activation (6–8), mitofusin 2 (*MFN2*), a mitochondrial fusion protein critical for energy homeostasis of the growing oocyte (9–11), and the apoptotic mediators, B-cell lymphoma 2 (*BCL2*) and BCL2-associated X (*BAX*), are recognized as some of the key determinants of the viability and competence of oocytes and developing embryos (12).

Oocytes are sourced from abattoir-derived ovaries or from hormonally synchronized live donors via laparoscopic ovum pick-up (LOPU). Abattoir ovaries are inexpensive and readily available, but are subject to uncontrolled donor variability and post-mortem ischemic damage (13). LOPU, in contrast, enables repeated recovery from live elite female goats and provides physiologically staged oocytes of potentially superior quality; however, the technology is not widely used in practice for goat production due to the complexity of the procedures (14, 15). Most of the comparative studies in goats have primarily focused on embryo yields. We conducted a controlled comparative study in bakerwal goats to evaluate the oocyte yield, oocyte quality, IVEP outcomes, and expression profiles of *ZAR1, MFN2, BAX*, and *BCL2* genes in LOPU- versus abattoir-derived oocytes at specific stages during the *in-vitro* development process. This integrative approach provides mechanistic insights into the impact of oocyte source on developmental competence and gene expression profile in high-genetic-merit goats.

## 2 Materials and methods

### 2.1 Ethical approval

**Figure 1:**
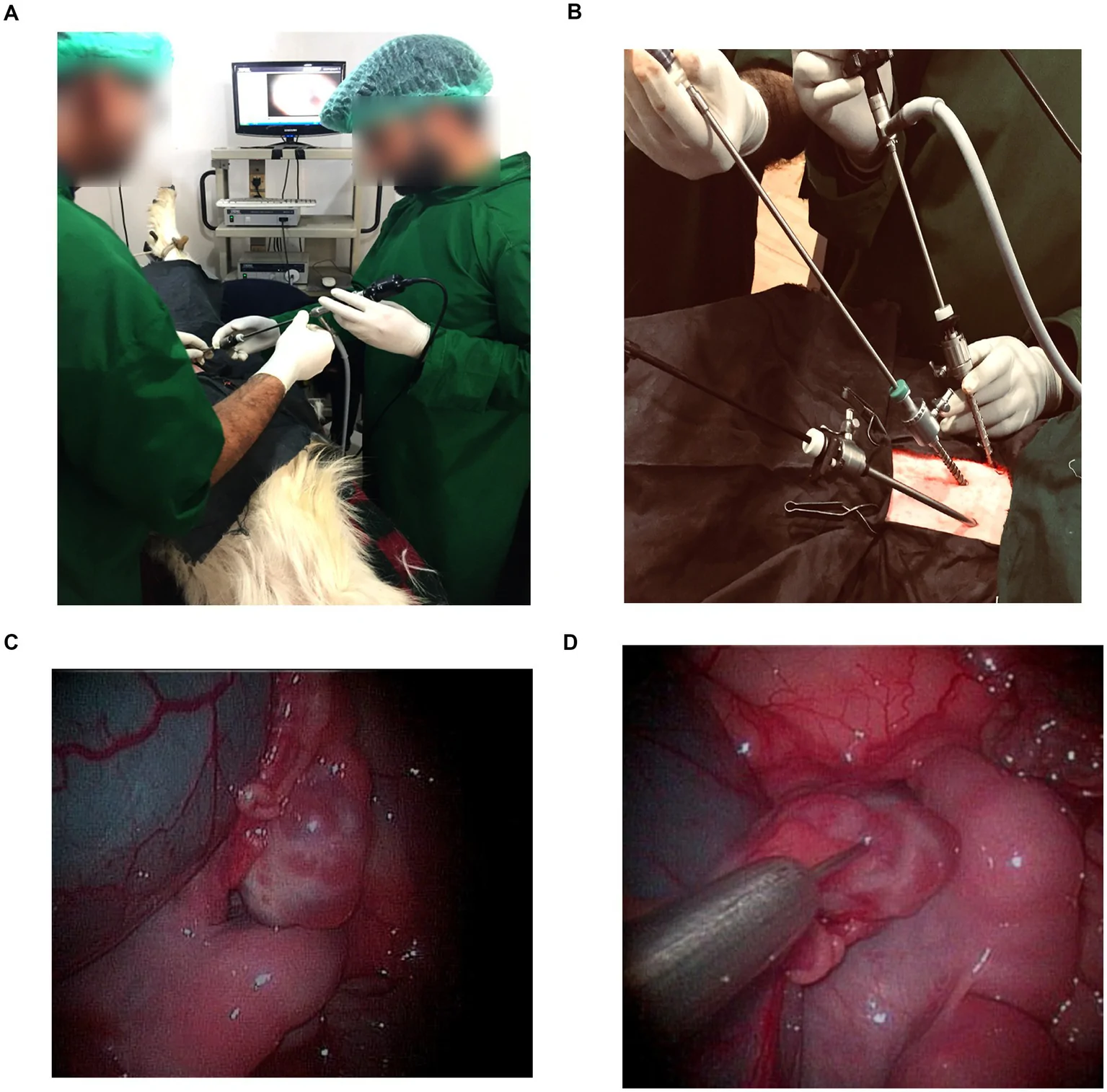
**(A)** Laparoscopic oocyte retrieval in goats. The animal was restrained in dorsal recumbency, and a three-port laparoscopic technique was used for follicular aspiration. **(B)** Outer view of the three-port laparoscopic setup showing the trocars, laparoscope, and aspiration needle inserted through the abdominal wall for follicular puncture. **(C)** The ovarian surface with visible follicles during oocyte retrieval through the laparoscope. **(D)** Laparoscopic view showing follicular aspiration being performed using a stainless-steel aspiration needle under direct visualization.

All animal procedures were approved by the Institutional Animal Ethics Committee (IAEC), Sher-e-Kashmir University of Agricultural Sciences and Technology of Kashmir, and conducted in accordance with the Committee for the Purpose of Control and Supervision of Experiments on Animals (CPCSEA) guidelines. For Figure 1A, identifiable human features have been anonymized. The ethics committee approval also covered the use and publication of procedural figures obtained during the study. Ovaries collected post-mortem from abattoirs were obtained as slaughter by-products; no animals were sacrificed specifically for this study. Reporting followed the ARRIVE 2.0 guidelines (16).

### 2.2 Animal management and superovulation

Twelve healthy Bakerwal does (aged 18–24 months), with a body condition score (BCS) ranging from 2.5 to 4.0 (scale 1–5), were selected for this study. All animals were maintained under uniform nutritional and husbandry conditions at the university farm. Before enrollment, reproductive status was assessed via transrectal ultrasonography (MyLab™ 40 Vet, Esaote, Genoa, Italy) equipped with linear and sector transducers (3.5–12 MHz) at the Veterinary Clinical Complex, SKUAST-Kashmir. Animals were confirmed non-pregnant (absence of gestational sacs) and cyclic, evidenced by the presence of a functional corpus luteum (>5 mm) and/or preovulatory follicles.

Estrus synchronization and superovulation were induced using an intravaginal progesterone protocol supplemented with gonadotropins. On Day 0, each doe received an intravaginal sponge impregnated with 45 mg fluorogestone acetate (Avikesil-S; ICAR-CSWRI, Avikanagar, India). On Day 9, superovulatory treatment was initiated with a single intramuscular injection of 750 IU equine chorionic gonadotropin (eCG; Folligon, MSD Animal Health), concurrent with luteolysis induction using 125 μg prostaglandin F₂*α* (PGF₂α; Lutalyse, Zoetis, USA). The intravaginal sponges were removed on Day 11, exactly 24 h before the scheduled Laparoscopic Ovum Pick-Up (LOPU) procedure (17).

### 2.3 Experimental design

We evaluated two oocyte sources: Oocytes obtained from abattoir-derived ovaries and oocytes retrieved from hormonally primed live donor goats via laparoscopic ovum pick-up (LOPU). In total, 1,517 cumulus–oocyte complexes (COCs) were retrieved from 338 abattoir ovaries, while 528 COCs were retrieved from 12 live donor goats (three LOPU sessions each). Retrieved COCs were graded as per the guidelines of the International Embryo Transfer Society into grades 1&2 to 3 & 4 (18). Only Grade 1 &2 oocytes were selected for IVEP. Developmental competence was assessed following *in-vitro* maturation (IVM) and *in-vitro* fertilization (IVF), with embryos cultured to the blastocyst stage. Parallel samples at the germinal vesicle (GV), metaphase II (MII), and blastocyst stages were collected for gene expression analysis.

### 2.4 Laparoscopic ovum pick-up

Donor goats were fasted for 36 h (feed) and 24 h (water) before laparoscopy. The surgical field, located cranial to the udder, was shaved and disinfected. Animals were allowed to rest undisturbed for 30 min, during which baseline physiological parameters (T0) were recorded. Preoperative medication consisted of ceftriaxone (10 mg/kg, IM; Intacef, Intas Pharmaceuticals Ltd., India) and meloxicam (0.3 mg/kg, IM; Melonex, Intas Pharmaceuticals Ltd., India), administered 30 min before surgery. Sedation was induced with xylazine–ketamine (46), followed by epidural anesthesia using 2% lignocaine hydrochloride (0.22 mL/kg; Lox, Neon Laboratories Ltd., India) (19).

Animals were positioned in dorsal recumbency at a 35 to 45° angle (Trendelenburg position) (20) to reduce the risk of visceral injury during trocar insertion (Figure 1A). Pneumoperitoneum was established with a veress needle using filtered air. Three trocar ports (Figure 1B), spaced 5–6 cm apart along the midline, were created. A 5-mm laparoscope (Karl Storz, Germany) was introduced through the central port. A second trocar allowed insertion of atraumatic grasping forceps for manipulation of the uterus and ovaries (Figure 1C), and the third port was used for the aspiration needle (Figure 1D). Lidocaine (2%) was locally infiltrated at each trocar site (19). Under laparoscopic guidance, the bladder and uterus were identified, and the ovaries were located. Ovaries were stabilized with grasping forceps and rotated to expose all visible follicles. Follicles with a diameter ≥2 mm were aspirated using a 20-gauge ovum pick-up needle into sterile 15 mL collection tubes (BD Falcon, Corning, NY, USA). The collection tubes were prefilled with warm aspiration medium (TCM-199 + heparin (10 IU/mL) + 0.3% bovine serum albumin). The collected follicular fluid was dispensed into 60 mm dishes (BD Falcon, Corning, NY, USA) and examined under a stereomicroscope for cumulus–oocyte complexes (COCs) (1). The circuit was periodically flushed with heparinized medium to prevent clot formation. Physiological monitoring during the procedure was performed using an oscillometric multiparameter monitor (SCURE Veterinary Monitor, CMS 8000 VET, Contec Medical Systems Co., Ltd., China). The port incisions were treated with a topical formulation containing gamma benzene hexachloride, proflavine, and cetrimide (Lorexane, Virbac Animal Health Pvt. Ltd., India). The preoperative antibiotic and analgesic regimen was continued for three consecutive days post-surgery, and antiseptic management of the incision site was maintained for 7 days postoperatively. Across three successive LOPU sessions, no significant differences were observed in follicular development or oocyte quality.

### 2.5 Abattoir ovary collection and COC recovery

**Figure 2:**
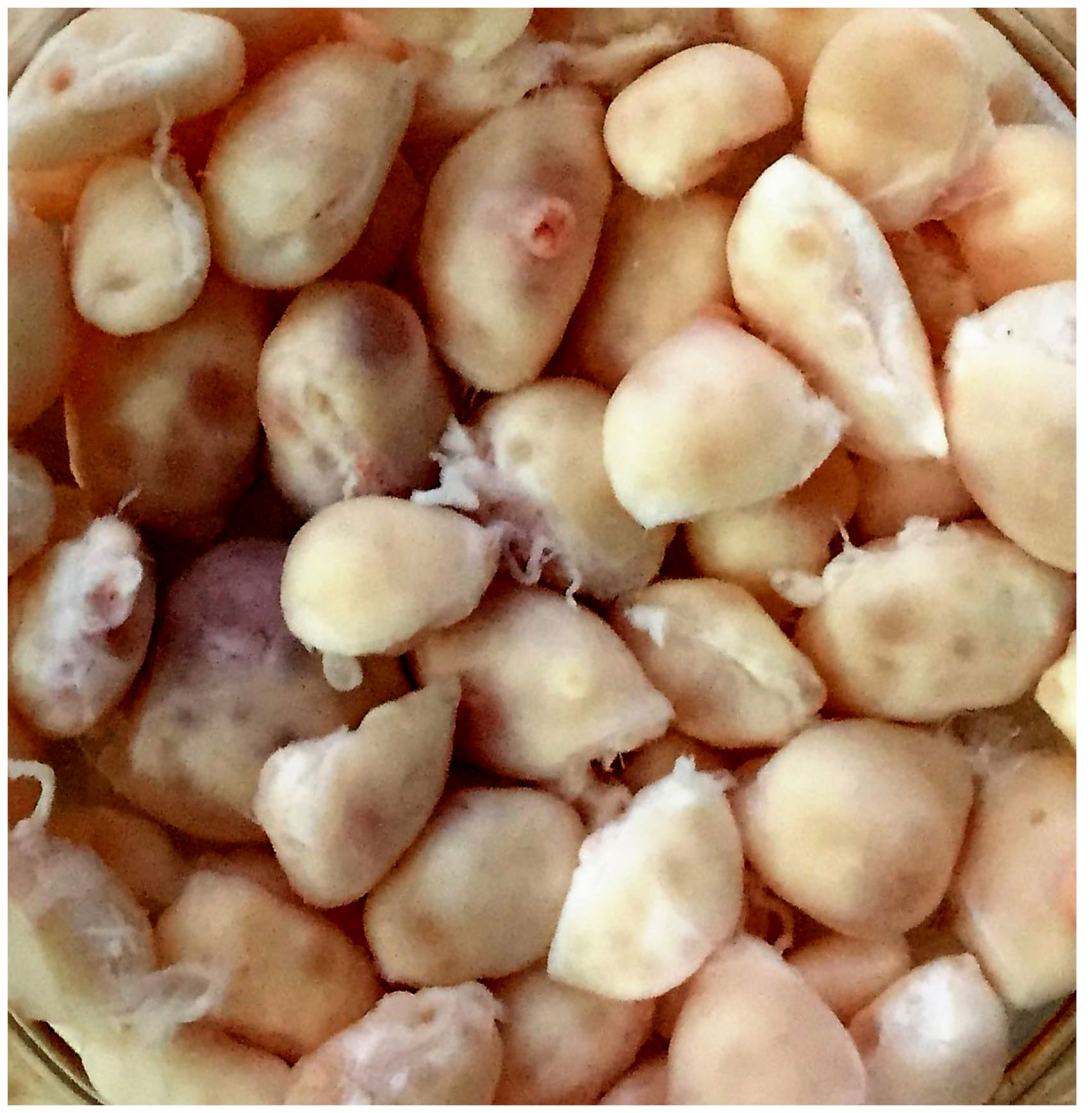
Slaughterhouse-derived goat ovaries.

Goat ovaries were collected from local slaughterhouses and rinsed 3–4 times in antibiotic-supplemented (400 IU/mL penicillin, 500 μg/mL streptomycin) warm saline (32–37 °C). They were transported to the laboratory in a thermos flask containing warm saline within 2–3 h of slaughter. In the lab, ovaries were again washed twice with antibiotic-supplemented saline, trimmed of extraneous tissue, and rewashed (Figure 2). Healthy ovaries were selected, and cumulus-oocyte complexes (COCs) were recovered by puncturing surface follicles (2–5 mm) with an 18-gauge needle in oocyte collection medium (Calcium & Magnesium free -DPBS + 0.3% BSA + 50 μg/mL gentamicin sulfate).

### 2.6 *In-vitro* maturation

The usable quality COCs from both sources were searched and selected under a zoom stereomicroscope (OLYMPUS, Japan, Model SZX 7 and SZX 10) in 100 mm x 100 mm square petri-dishes with a 13 mm grid. Oocytes were then transferred to 35 mm petri-dishes containing the washing medium (Hepes-buffered TCM-199 supplemented with 10% fetal bovine serum (FBS), 0.68 mM L-glutamine, 0.8 mM sodium pyruvate, and 50 μg/mL gentamicin sulfate). The selected oocytes were washed thrice with washing medium, followed by two washes in IVM medium (washing medium supplemented with 5 μg/mL pFSH, 1 μg/mL 17β-estradiol, 1 μg/mL LH, and 10% 52 Follicular fluid). Groups of 20–25 COCs were cultured in IVM medium. Cultures were performed in 80–100 μL IVM medium droplets under mineral oil at 38.5 °C in 5% CO₂ and 95% humidity for 26–27 h. The cumulus expansion and extrusion of the first polar body confirmed nuclear maturation.

### 2.7 *In-vitro* fertilization

Fresh semen was collected from a single selected buck housed at the university farm by artificial vagina method in sterile 15 mL tubes maintained at 37 °C. The collected semen was transported to the lab within 5–10 min at 37 °C. 200 μL of the freshly collected semen was placed at the bottom of a fresh 15 mL tube containing 5 mL of swim-up medium (TCM199 medium without BSA and heparin) for swim-up of actively motile spermatozoa. The tube was kept undisturbed for 30 min in a CO_2_ incubator, and the topmost 4 mL supernatant was collected carefully and transferred to a fresh 15 mL tube containing 6 mL swim-up medium and centrifuged at 1200 rpm for 5–6 min. The pellet was dissolved in Capacitation medium (TCM199 medium with 6 mg/mL BSA and 10 μg/mL heparin) and incubated in CO_2_ incubator for 45–60 min for capacitation. Simultaneously, the COCs with expanded cumulus oophorous were partially denuded using hyaluronidase enzyme (1 mg/mL) and incubated at 38.5 °C in a CO_2_ incubator for two to three minutes. The cumulus layers surrounding the COCs were removed by gentle pipetting, and the denuded oocytes were washed in TCM 199 medium supplemented with 6 mg/mL BSA. After two to three washings, the oocytes are distributed in groups of 15 to 20 oocytes in 100 μL drops of fertilization medium (TCM 199 medium supplemented with 6 mg/mL BSA). The final sperm concentration was determined using the Bürker chamber, and a final concentration of 10^6^ spermatozoa per milliliter was used for IVF.

### 2.8 Embryo culture

After 18 h of co-incubation, presumed zygotes were washed in washing medium to remove spermatozoa by gentle pipetting, and were cultured in groups of 15–20 in 100 μL droplets of G1 embryo culture medium for 48 to 72 h. After 72 h, the G1 embryo culture medium was replaced by G2 medium and cultured for up to 7–8 days in an incubator at 38.5 °C, 5% CO₂, 5% O₂, 90% N₂ (Thermo-Fisher Scientific, USA, Model 3,131). The cleavage rate was recorded 24 h after co-incubation, in terms of oocytes that cleaved to the 2-cell stage or beyond. The developmental stages were recorded on day 8. Activated oocytes were cultured in G1/G2 embryo culture media in the same way.

### 2.9 Quantitative transcript analysis

Pools of 15 oocytes or embryos from each developmental stage were collected per replicate for transcript analysis. Total RNA was extracted using the *Arcturus PicoPure RNA Isolation Kit* (Applied Biosystems) according to the manufacturer’s instructions. RNA concentration and purity were assessed using a *NanoDrop 2000* spectrophotometer (Thermo Scientific, Waltham, MA, USA). Only samples with RNA yields ≥ 0.5 μg/μL and A260/280 ratios between 1.9 and 2.0 were used for cDNA synthesis. To eliminate any genomic DNA contamination, RNA samples were treated with *DNase I* (Sigma-Aldrich, USA) before reverse transcription. Equal amounts of RNA (85 ng) were reverse transcribed into first-strand cDNA using the *RevertAid First Strand cDNA Synthesis Kit* (Thermo Scientific) with oligo (dT) primers. Negative controls lacking reverse transcriptase were included to confirm the absence of genomic DNA. Synthesized cDNA was stored at −20 °C until further use. Transcript levels of *ZAR1*, *MFN2*, *BAX*, and *BCL2* were quantified in germinal vesicle (GV) oocytes, *in vitro*–matured (MII) oocytes, and blastocysts, each in three replicates. Quantitative PCR reactions (20 μL) contained 10 μL of 2 × SYBR Green Master Mix (Thermo Scientific), 1 μL of cDNA, and 0.2 μL of each primer (10 μM). Primer sequences, designed using Primer3 software, are listed in Table 1. qPCR was performed on a *Roche LightCycler® 480 II* system using SYBR Green I detection chemistry. Expression data were normalized to the geometric mean of the housekeeping gene *GAPDH*. Relative expression levels were calculated using the comparative Ct method (2^–ΔΔCt) as described by (21), where:

- ΔΔC_T_ = ΔC_T (sample)_ ˗ ΔC_T (calibrator)_
- ΔC_T (sample) =_ {C_T (target gene)_ ˗ C_T (reference gene)_} _sample_
- ΔC_T (calibrator) =_ {C_T (target gene)_ ˗ C_T (reference gene)_} _calibrator_

**Table 1:** Primer details of ZAR1, MFN2, BAX, BCL2, and GAPDH genes.

Name of the gene	Primer sequence		Size of amplicon
ZAR1	Forward primer	CTCCTCCTTTTCTGCTGCTC	117 bp
	Reverse primer	ACAGGCTCTCCTACGCATTT	
MFN2	Forward primer	GACCGCATCTTCTTCGTGT	150 bp
	Reverse primer	GCACTCCTCAAATCTCCTCTC	
BAX	Forward primer	TGGATGACCGAGTACCTGAA	121 bp
	Reverse primer	CAGCCAGGAGAAATCAAACA	
BCL2	Forward primer	AGTGGCGGCTGAAATGTT	120 bp
	Reverse primer	AGTAGAAAAGGGCGACAACC	
GAPDH	Forward primer	GCACAGTCAAGGCAGAGAAC	101 bp
	Reverse primer	ACCAGCATCACCCCACTT	

### 2.10 Statistical analysis

Data analyses were performed using SPSS software (version 27.0; IBM Corp., Armonk, NY, USA). Before analysis, all percentage data regarding developmental competence (maturation, cleavage, morula, and blastocyst rates) were subjected to arcsine transformation to normalize the data distribution and satisfy the assumptions of parametric testing. One-way Analysis of Variance (ANOVA) was subsequently used to compare the means of developmental parameters and relative gene expression levels (RQ values) between the experimental groups. Fisher’s Least Significant Difference (LSD) post-hoc test was employed to determine specific differences between the LOPU-derived and abattoir-derived groups, as the study design prioritized planned pairwise comparisons. All data are presented as mean ± standard error of the mean (SEM). Differences were considered statistically significant at *p* < 0.05.

## 3 Results

### 3.1 Comparison of oocyte yield and quality

A total of 1,517 COCs were recovered from 338 abattoir ovaries, averaging 4.5 ± 0.7 per ovary, of which 75.3% were Grade 1 & 2. By contrast, 528 COCs were obtained from 12 hormonally synchronized goats via LOPU, corresponding to 7.3 ± 0.4 per ovary, with 82.3% classified as Grade 1 & 2 (Table 2). Thus, while abattoir ovaries provided higher absolute numbers, LOPU yielded a significantly greater proportion of developmentally competent oocytes.

**Table 2:** Comparison of percentages of usable quality oocytes (grade 1 & 2) retrieved.

Source of ovaries	Total COCs obtained (*n*)	Usable COCs (Grade 1&2) (*n*)	Percentage of Usable COCs (Mean ± SEM)
Live animal (LOPU)	528	435	82.3 ± 2.89ᵃ
Abattoir	1,517	1,141	75.3 ± 2.09ᵃ

### 3.2 Comparison of *in vitro* maturation

**Figure 3:**
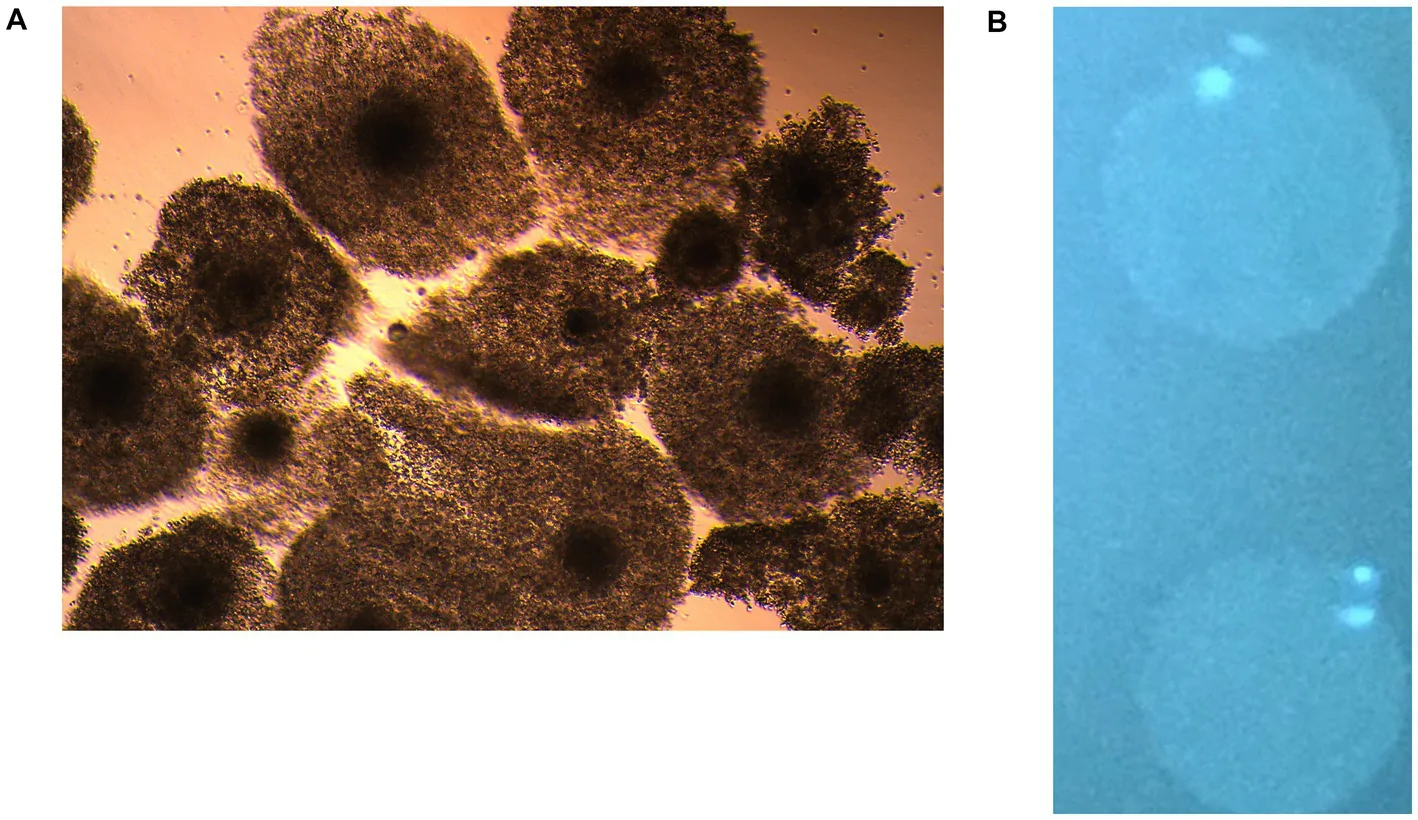
**(A)** *In vitro* matured oocytes with cumulus cell mass showing expansion under culture conditions, indicating successful oocyte maturation. **(B)** *In vitro* matured oocytes stained with bisBenzimidine stain showing the extruded polar body, indicating successful nuclear maturation.

After *in-vitro* maturation, 1,141 abattoir-derived and 435 LOPU-derived COCs were assessed (Table 3). Maturation rates were not significantly different between sources (abattoir: 76.3%; LOPU: 81.1%; *p* > 0.05), indicating that both sources provided adequate support for nuclear maturation (Figures 3A,B).

**Table 3:** Comparison of maturation percentage between the groups.

Source of oocytes	Oocytes put to maturation (*n*)	Oocytes matured (*n*)	Maturation percentage (Mean ± SEM)
Live animal (LOPU)	435	353	81.1 ± 1.2ᵃ
Abattoir	1,141	871	76.3 ± 2.1ᵃ

### 3.3 Comparison of *in-vitro* fertilization

**Figure 4:**
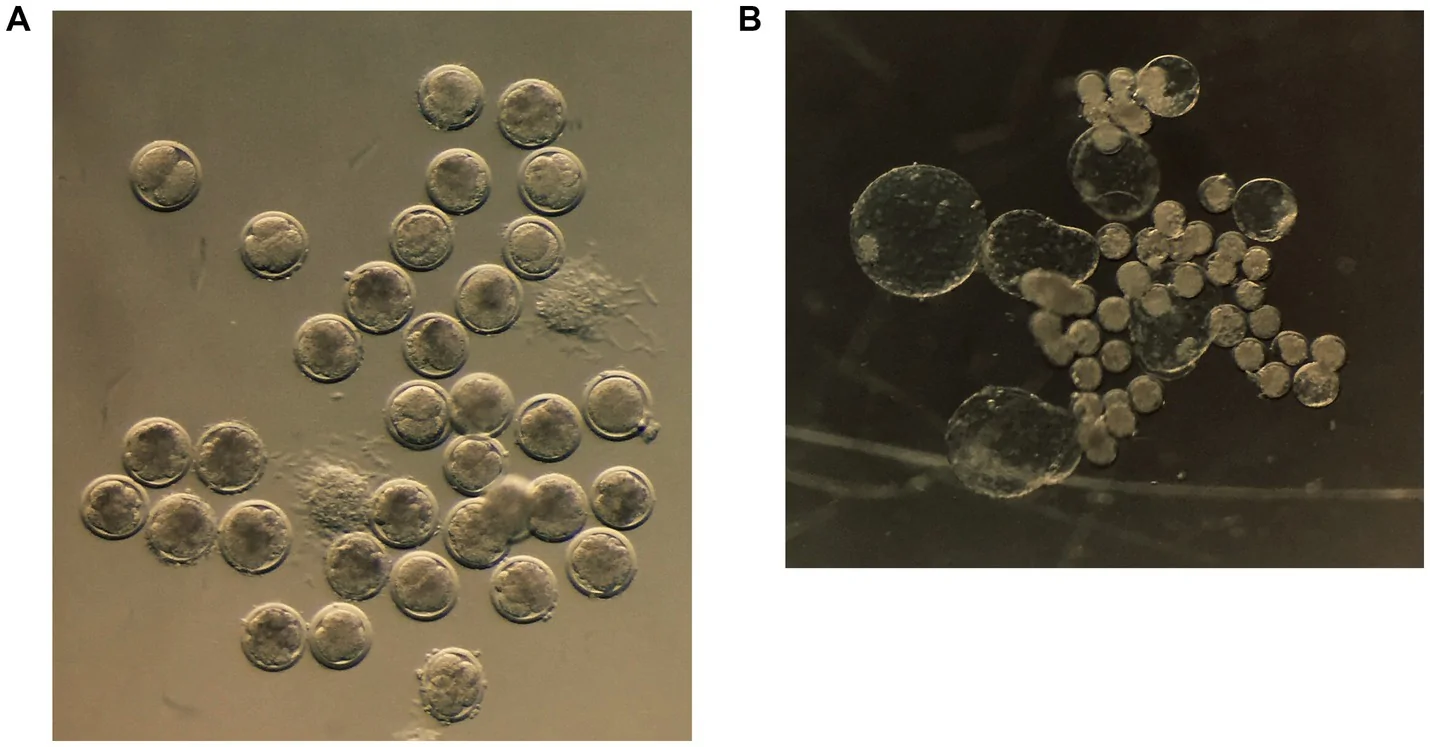
**(A)** Cleaved caprine oocytes at different stages of development following *in vitro* fertilization. **(B)** *In vitro*-produced caprine blastocysts following fertilization and culture.

Following IVF, cleavage rates were significantly higher in LOPU-derived oocytes than abattoir-derived oocytes (54.9% vs. 45.2%; *p* < 0.05). Similarly, progression to the morula stage was greater in the LOPU group (45.9% vs. 34.9%, p > 0.05), as was the blastocyst yield (29.5% vs. 19.0%; p < 0.05) (Table 4). Overall, LOPU oocytes demonstrated a significant increase in blastocyst formation compared with abattoir counterparts (Figures 4A,B).

**Table 4:** Comparison of *in vitro* development between the groups.

Source of oocytes	Treatment	Oocytes treated (*n*)	Cleavage % (*n*) (Mean ± SEM)	Morula % (*n*) (Mean ± SEM)	Blastocyst % (*n*) (Mean ± SEM)
Live animal (LOPU)	IVF	222	(122) 54.9 ± 3.0ᵃ	(56) 45.9 ± 1.9ᵃ	(36) 29.5 ± 3.0ᵃ
Abattoir	IVF	425	(192) 45.2 ± 4.2^b^	(67) 34.9 ± 1.7ᵃ	(37) 19.0 ± 2.0^b^

The superscript letters “a” and “b” indicate statistically significant differences between values bearing different superscripts. Values sharing the same superscript are not significantly different.

### 3.4 Comparison of gene expression dynamics

**Figure 5:**
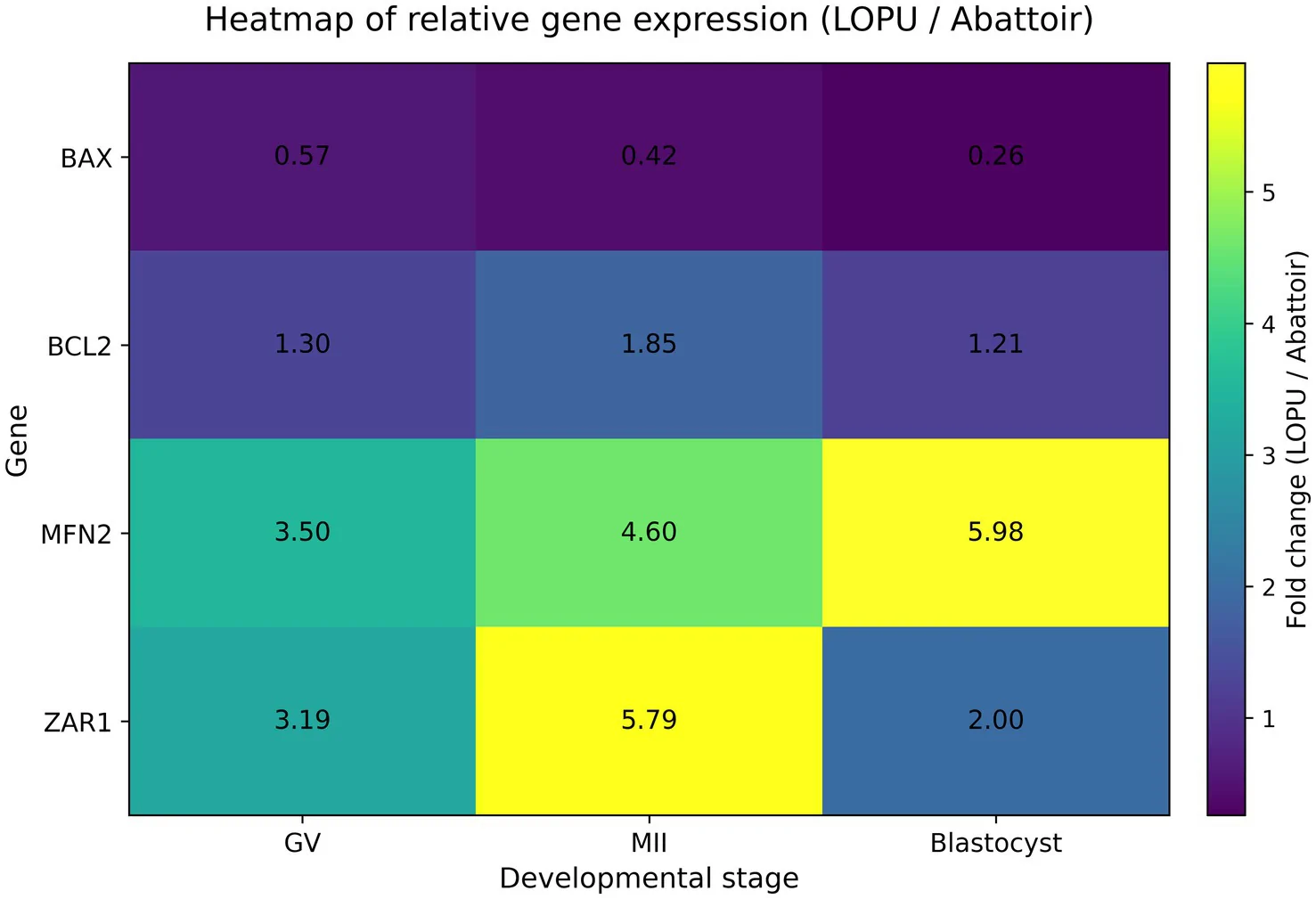
Heatmap of fold-change (Live ÷ Abattoir) across genes (*ZAR1*, *MFN2*, *BAX*, *BCL2*) and developmental stages (GV, MII, Blastocyst). *ZAR1* and *MFN2* were upregulated in LOPU, while *BAX* was downregulated and *BCL2* was moderately elevated.

**Figure 6:**
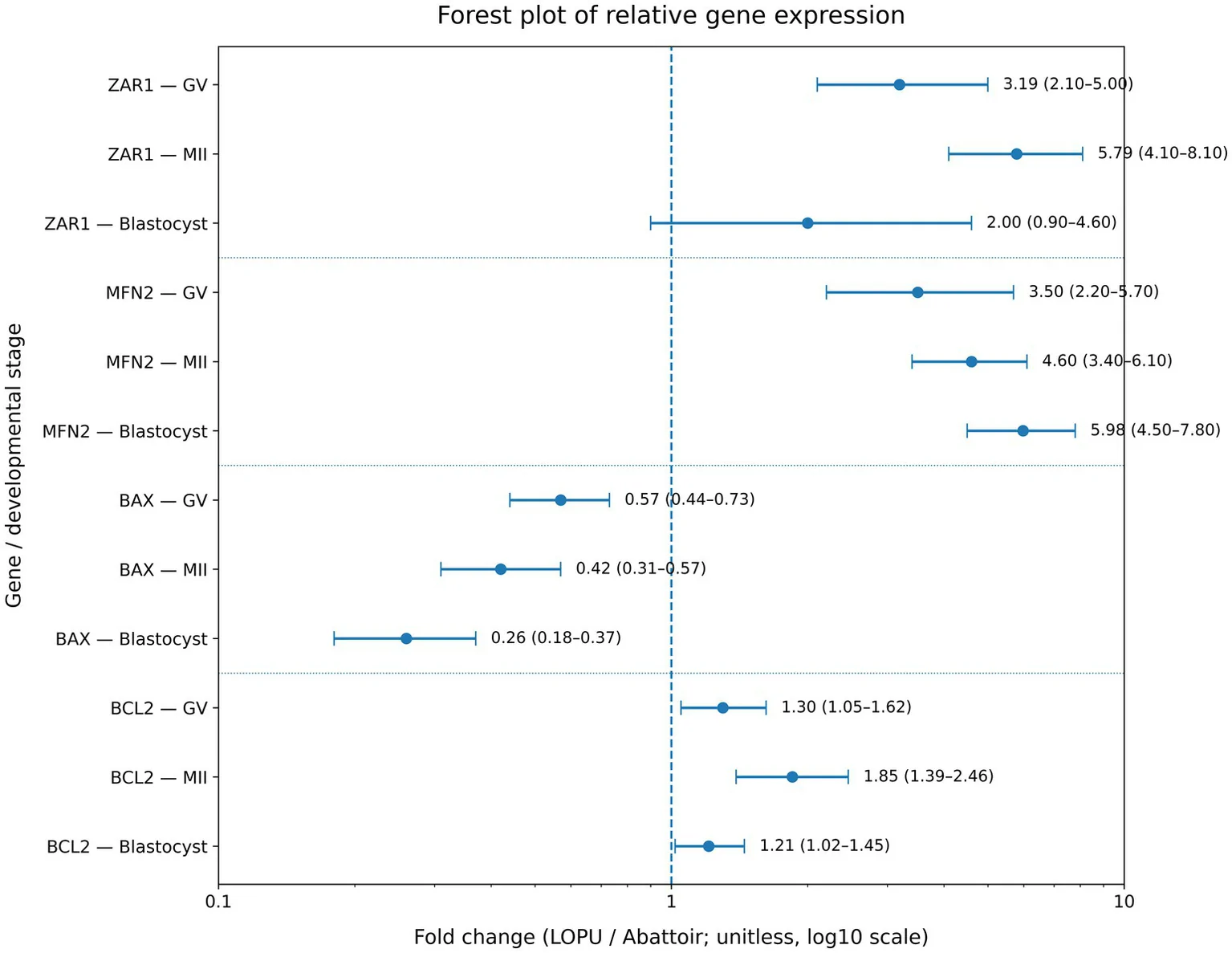
Forest plot of fold-change (Live ÷ Abattoir) for gene expression with 95% confidence intervals. Dashed vertical line at 1.0 indicates no difference. ZAR1 and MFN2 were significantly upregulated in LOPU oocytes, BAX was significantly downregulated, and BCL2 moderately increased across stages.

Comparison of relative gene expression (RQ, 2^–ΔΔCt^) between LOPU- and abattoir-derived oocytes revealed consistent stage- and gene-specific differences. Fold change estimates (Live ÷ Abattoir) indicated that ZAR1 expression was markedly higher in LOPU oocytes at the immature (3.19-fold, 95% CI 2.1–5.0) and mature stages (5.79-fold, 95% CI 4.1–8.1), declining toward equivalence at the blastocyst stage (2-fold, 95% CI 0.9–4.6), consistent with degradation of maternal transcripts after embryonic genome activation. MFN2 expression showed a progressive and consistent increase in LOPU embryos, with fold-changes ranging from 3.5-fold at the immature stage (95% CI 2.2–5.7) to nearly 5.98-fold at the blastocyst stage (95% CI 4.5–7.8), suggesting enhanced mitochondrial preparedness. In contrast, BAX expression was consistently lower in LOPU embryos (0.26–0.57 fold vs. abattoir), indicating reduced apoptotic priming. The expression of BCL2, a pro-survival mediator, was higher in LOPU embryos, particularly at MII (1.85-fold, 95% CI: 1.39–2.46) and remained moderately increased at the blastocyst stage (1.21-fold, 95% CI: 1.02–1.45). To visualize patterns, a heatmap highlighted the consistent upregulation of *ZAR1, MFN2,* and *BCL2*, and downregulation of BAX, across developmental stages in LOPU oocytes and embryos (Figure 5). A forest plot illustrated fold-change estimates demonstrating that differences in *ZAR1, MFN2*, and *BAX* were robust, while *BCL2* showed moderate but significant elevation. It highlights that LOPU-derived oocytes exhibit a favorable transcriptomic signature characterized by stronger maternal programming (*ZAR1*), enhanced mitochondrial dynamics (*MFN2*), and a shifted apoptotic balance (lower *BAX/BCL2* ratio), consistent with their superior developmental competence (Figure 6).

## 4 Discussion

This study provides an integrative comparison of LOPU- and abattoir-derived oocytes in bakerwal goats, coupling developmental outcomes with molecular signatures of competence. While both sources support *in-vitro* embryo production (IVEP), LOPU-derived oocytes consistently exhibit higher developmental progression, improved morphology, and favorable gene-expression profiles. The superior cleavage, morula, and blastocyst yields observed in LOPU-derived oocytes align with previous reports in goats, sheep, buffalo, and gilts, where oocytes retrieved via LOPU from synchronized donors demonstrated enhanced competence compared with abattoir sources (1, 13, 18). The disparity likely reflects intrinsic differences in follicular environments: LOPU oocytes originate from follicles exposed to controlled hormonal stimulation, whereas abattoir oocytes derive from donors of unknown age, physiological status, and ante-mortem stress history. Additional ischemic delays during ovary transport exacerbate cytoplasmic compromise in abattoir-derived oocytes (1, 12). Thus, the advantage of LOPU is not merely numerical but biologically programmed.

### 4.1 Molecular correlates of competence

Gene expression profiling suggests a mechanistic association underlying the developmental disparity between oocytes of different origins. The maternal-effect gene Zar1, which plays a crucial role in oocyte maturation and activation of early embryonic development (7, 8), was significantly upregulated in LOPU-derived oocytes at both the GV and MII stages. Recent studies indicate that Zar1 stabilizes maternal transcripts in GV-stage oocytes by promoting the formation of a mitochondria-associated, membraneless compartment that sequesters maternal mRNAs (6). This structural role is critical; recent findings in human and bovine models indicate that variants disrupting ZAR1’s ability to form MARDOs lead to complete meiotic arrest and infertility (22). This higher Zar1 expression in LOPU-derived oocytes, therefore, implies a stronger reservoir of maternal programming factors, which may contribute to more reliable zygotic genome activation (ZGA) (8). During normal embryogenesis, maternal mRNAs are progressively degraded and replaced by embryonic transcripts, enabling meiotic cell-cycle progression and early development (23). Proteasomal degradation of ZAR1 protein initiates disassembly of the mitochondria-associated compartment, facilitating timely maternal mRNA turnover (6). Accordingly, the reduced Zar1 expression observed at the blastocyst stage likely reflects this physiological clearance accompanying the maternal-to-zygotic transition, which occurs at the 8-cell stage in goats (24, 25).

### 4.2 Mitochondrial dynamics and competence

Mitofusin 2 (MFN2), a key regulator of mitochondrial fusion (26, 27), showed progressive upregulation across developmental stages, peaking in early blastocysts from LOPU embryos. MFN2, a mitochondrial outer-membrane GTPase, mediates mitochondrial fusion and maintains contact with the endoplasmic reticulum (ER), ensuring mitochondrial integrity, energy production, and calcium balance (28–32). These contact sites are essential for ensuring mitochondrial integrity, ATP production, and calcium homeostasis (11, 33). Although many findings derive from mice (10, 34) and bovine (27), mechanistic parallels apply across mammals. Loss of MFN2 leads to mitochondrial dysfunction, reduced membrane potential, ATP depletion, and abnormal Ca^2+^ oscillations (9), all of which are detrimental to meiotic progression and early embryogenesis (10, 28). In ruminants, strong *MFN2* activity supports balanced energy distribution and protects against the oxidative stress typical of *in vitro* culture. Higher MFN2 expression has been consistently linked with better oocyte quality and developmental competence across species (29, 32). The higher *MFN2* expression observed in LOPU embryos likely reflects enhanced “mitochondrial readiness,” facilitating the high energy demands of compaction and blastulation. Conversely, the reduced expression in abattoir oocytes mirrors the “aged” oocyte phenotype described in recent stress-response studies (9, 35), suggesting that enhancing *MFN2* expression through NAD^+^ precursors (36) or optimized culture systems could be a pathway to improve yields from compromised sources (45).

#### 4.2.1 Apoptotic balance

The apoptotic balance, as indicated by the BAX/BCL2 ratio, further differentiates the developmental potential of oocytes derived from different sources. Abattoir-derived embryos displayed elevated pro-apoptotic BAX and reduced pro-survival BCL2 expression, a molecular signature often linked to early embryonic arrest and fragmentation (37, 38). This imbalance suggests a higher propensity for apoptosis and reduced embryo viability, likely triggered by the ante-mortem stress and ischemia associated with slaughterhouse recovery (1). In contrast, LOPU-derived embryos maintained lower BAX and higher BCL2 expression, indicating a more favorable cellular environment less prone to apoptotic signaling. These findings align with previous studies demonstrating that a favorable apoptotic profile (high BCL2/BAX ratio) contributes to improved oocyte quality and embryo development (39, 40). Furthermore, *BCL2* family proteins have recently been shown to safeguard mitochondrial integrity during maturation, preventing the cytochrome c release that triggers fragmentation (41). Collectively, these gene expression dynamics emphasize the molecular superiority of LOPU-derived oocytes, which appear to be more resilient and better suited for successful early-stage development.

### 4.3 Morphological correlates and biotechnological implications

From an applied perspective, abattoir ovaries remain invaluable for training, high-throughput IVEP, and exploratory research where quantity outweighs quality. However, for advanced biotechnologies such as CRISPR/Cas9-mediated gene editing and somatic cell nuclear transfer (SCNT), the predictability and cytoplasmic competence of the oocyte are critical. The apparent low cost of abattoir material is often offset by lower efficiency rates when applied to advanced biotechnologies. Regarding the conservation of wild species, post-mortem recovery remains the only practical option when an animal dies unexpectedly or is culled (42). However, reliance solely on opportunistic post-mortem recovery is risky due to variable ischemic intervals and unknown health status (43). For managed populations of endangered species, LOPU represents a superior strategy. Recent studies affirm that LOPU is one of the safe and effective methods for obtaining high-quality oocytes from wild felids, such as pumas and jaguars (44). Therefore, LOPU allows for the establishment of “safety biobanks” with high-competence embryos before an animal reaches senescence or dies, complementing post-mortem efforts rather than replacing them. For precision interventions where the genetic value of the donor is high, LOPU positions itself as the requisite method to ensure the highest probability of live offspring.

### 4.4 Limitations and future perspectives

The findings of this study are supported by the integration of developmental, morphological, and molecular endpoints, utilizing a repeated-measures design that accounted for donor and batch effects. However, limitations remain. The study was restricted to a targeted gene panel, and functional assays (e.g., mitochondrial membrane potential, ROS levels, or apoptotic indices) were not performed. Additionally, the ultimate test of competence, live birth following embryo transfer, was outside the scope of this study. Future investigations should aim to bridge this gap by incorporating single-cell RNA sequencing (scRNA-seq) to capture the heterogeneity of transcriptomic landscapes, alongside embryo transfer trials to confirm that the molecular superiority of LOPU oocytes translates into higher kidding rates.

## 5 Conclusion

This study highlights that while abattoir-derived ovaries yield a greater number of oocytes, LOPU-derived oocytes possess a distinct developmental and molecular advantage characterized by enhanced maternal programming (*ZAR1*), mitochondrial readiness (*MFN2*), and apoptotic resistance (*BCL2*). These findings do not propose new superovulation regimens for goats but provide transferable molecular benchmarks (maternal-effect genes, mitochondrial dynamics, apoptotic balance) that can guide optimization of oocyte selection and *in vitro* culture strategies across ruminants. These findings have two practical implications for improving IVEP protocols: first, they establish *MFN2* and *ZAR1* as potential molecular biomarkers for selecting competent oocytes before high-cost procedures like cloning; and second, they suggest that culture media for abattoir-derived oocytes may require specific supplementation (e.g., mitochondrial antioxidants) to mimic the superior physiological state of LOPU oocytes. Thus, LOPU should be the method of choice for precision reproductive biotechnologies where embryo quality is paramount.

## Data Availability

The original contributions presented in the study are included in the article/supplementary material, further inquiries can be directed to the corresponding author.
