# Correction: Comparative developmental competence and gene expression in laparoscopically retrieved- and abattoir-derived goat oocytes

**DOI:** 10.3389/fvets.2026.1983590

**Published:** 2026-09-21

**Authors:** Abrar A. Malik, Riaz A. Shah, Syed Hilal Yaqoob, Mohammad Abrar Gayas, Nida Handoo, Suhail N. Magray, Mujeeb R. Fazili, Muneer A. Dar, Nafis I. Assad, Saba Nazir, Syed M. Shah, Nadeem Shabir, Syed M. Ahmad

**Affiliations:** 1 Embryo Biotech Laboratory, Division of Animal Biotechnology, Faculty of Veterinary Sciences, SKUAST-Kashmir, Srinagar, India; 2 Center of Excellence for Reproductive Biotechnology, Mountain Livestock Research Institute, SKUAST-Kashmir, Manasbal, India; 3 Department of Animal Husbandry, Govt of UT of Jammu and Kashmir, Srinagar, India; 4 Division of Teaching Veterinary Clinical Complex, Faculty of Veterinary Sciences, SKUAST-Kashmir, Srinagar, India; 5 Vaccine Biotechnology Laboratory, Division of Animal Biotechnology, Faculty of Veterinary Sciences, SKUAST-Kashmir, Srinagar, India; 6 Genomics Laboratory, Division of Animal Biotechnology, Faculty of Veterinary Sciences, SKUAST-Kashmir, Srinagar, India

**Keywords:** abattoir ovary, gene expression, goat, IVEP, LOPU, oocyte competence

There was a mistake in Table 1 as published.

The primer sequences for ZAR1 reverse and MFN2 forward were inadvertently duplicated. The error was confined to the reporting of the primer sequences in Table 1; the corrected sequences correspond to the primers actually used in the experiments, and therefore the underlying qPCR data and analyses remain unchanged.

The corrected Table 1 appears below.

**Table 1:** Primer details of ZAR1, MFN2, BAX, BCL2, and GAPDH genes.

Name of the gene	Primer sequence		Size of amplicon
ZAR1	Forward primer	CTCCTCCTTTTCTGCTGCTC	117 bp
	Reverse primer	ACAGGCTCTCCTACGCATTT	
MFN2	Forward primer	GACCGCATCTTCTTCGTGT	150 bp
	Reverse primer	GCACTCCTCAAATCTCCTCTC	
BAX	Forward primer	TGGATGACCGAGTACCTGAA	121 bp
	Reverse primer	CAGCCAGGAGAAATCAAACA	
BCL2	Forward primer	AGTGGCGGCTGAAATGTT	120 bp
	Reverse primer	AGTAGAAAAGGGCGACAACC	
GAPDH	Forward primer	GCACAGTCAAGGCAGAGAAC	101 bp
	Reverse primer	ACCAGCATCACCCCACTT	

There was a mistake in Table 4 as published.

The reported counts for morulae and blastocysts were incorrect, although the corresponding percentages were correct. The error was purely typographical and did not affect the underlying data, statistical analysis, results, or conclusions of the study.

The corrected Table 4 appears below.

**Table 4:** Comparison of *in vitro* development between the groups.

Source of oocytes	Treatment	Oocytes treated (*n*)	Cleavage % (*n*) (mean ±SEM)	Morula % (*n*) (mean ±SEM)	Blastocyst % (*n*) (mean ±SEM)
Live animal (LOPU)	IVF	222	(122) 54.9 ± 3.0^a^	(56) 45.9 ± 1.9^a^	(36) 29.5 ± 3.0^a^
Abattoir	IVF	425	(192) 45.2 ± 4.2^b^	(67) 34.9 ± 1.7^a^	(37) 19.0 ± 2.0^b^

The superscript letters “a” and “b” indicate statistically significant differences between values bearing different superscripts. Values sharing the same superscript are not significantly different.

There was a mistake in Figure 5 as published.

The heatmap was incorrectly generated during figure preparation, resulting in an inaccurate representation of the gene-wise fold-change patterns. The underlying gene-expression data and statistical analyses were correct and remain unchanged.

The corrected Figure 5 appears below.

**Figure 5:**
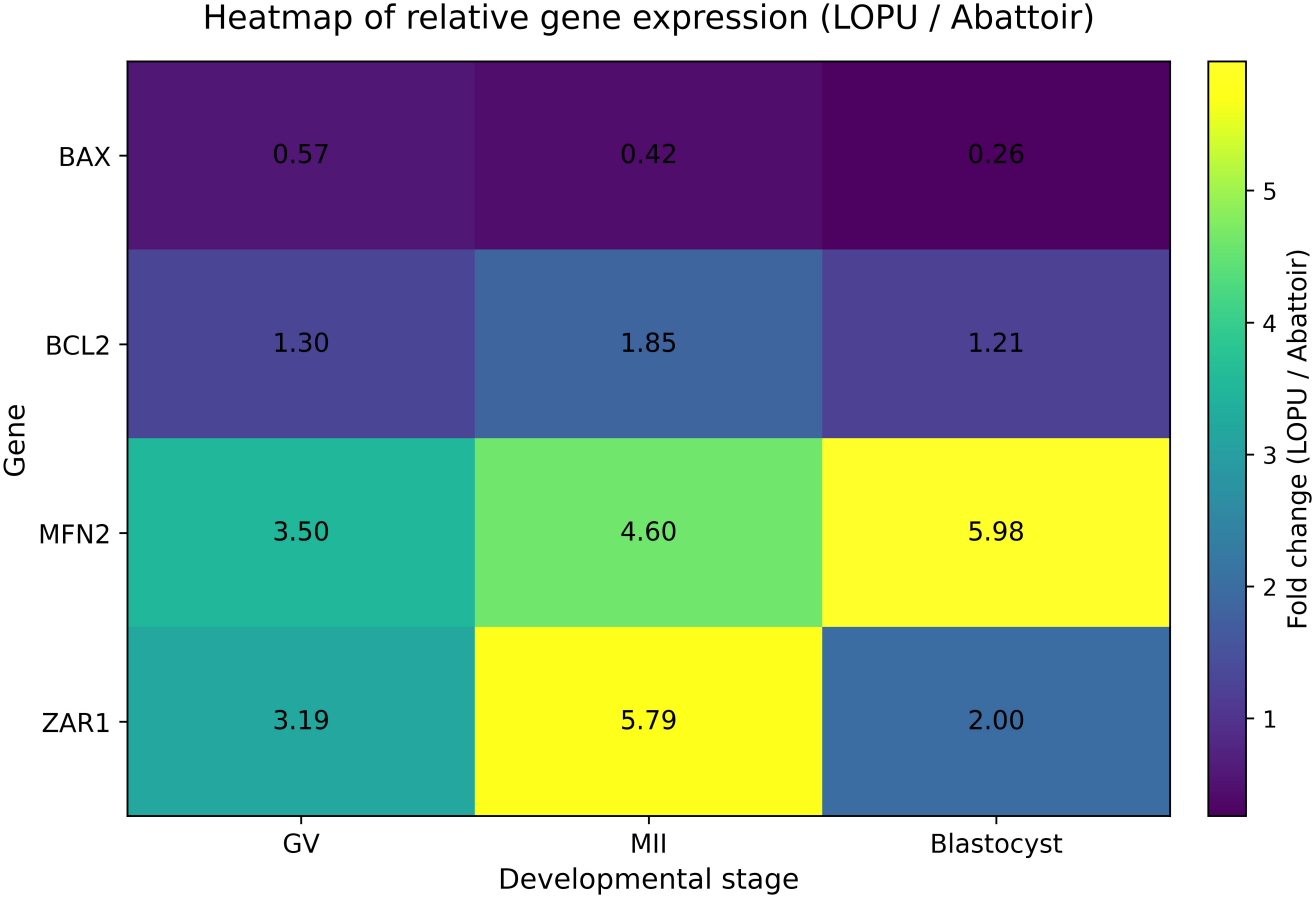
Heatmap of fold-change (Live ÷ Abattoir) across genes (ZAR1, MFN2, BAX, BCL2) and developmental stages (GV, MII, Blastocyst). ZAR1 and MFN2 were upregulated in LOPU, while BAX was downregulated and BCL2 was moderately elevated.

There was an error in Figure 6 as published.

The forest plot was incorrectly generated during the preparation of the figure, resulting in an inaccurate visual representation of the corresponding effect estimates and confidence intervals. The underlying data, statistical analyses, and reported results were correct and remain unchanged. The error was limited to the generation and presentation of the forest plot.

The corrected Figure 6 appears below.

**Figure 6:**
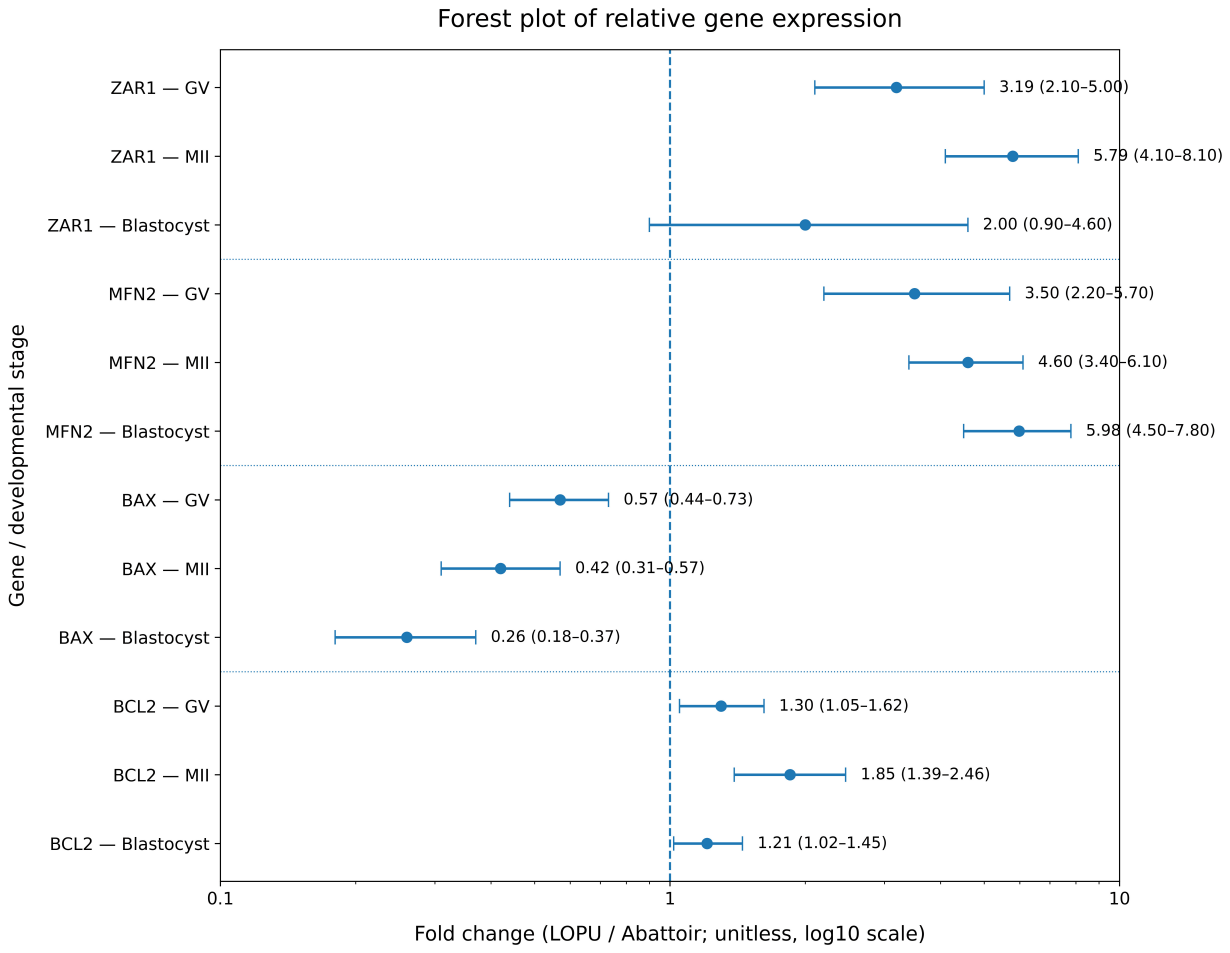
Forest plot of fold-change (Live ÷ Abattoir) for gene expression with 95% confidence intervals. Dashed vertical line at 1.0 indicates no difference. ZAR1 and MFN2 were significantly upregulated in LOPU oocytes, BAX was significantly downregulated, and BCL2 moderately increased across stages.

There was an error in the caption of Figure 5 as published.

The developmental stage was incorrectly described as “morula” instead of “blastocyst.”

The corrected caption of Figure 5 appears below:

“Heatmap of fold-change (Live ÷ Abattoir) across genes (ZAR1, MFN2, BAX, BCL2) and developmental stages (GV, MII, Blastocyst). ZAR1 and MFN2 were upregulated in LOPU, while BAX was downregulated and BCL2 was moderately elevated.”

The original version of this article has been updated.

